# Combined surgery and proton radiotherapy in the management of craniopharyngiomas: an update with paradigmatic and challenging case scenarios

**DOI:** 10.1007/s10143-026-04258-1

**Published:** 2026-03-31

**Authors:** Federico Valeri, Matteo Zoli, Sara Lillo, Francesco Tengattini, Francesca Colombo, Ernesto Pasquini, Gennaro Salierno, Alessandro Carretta, Luca D’Ambrosio, Daniel Babaei, Edoardo Agosti, Pier Paolo Mattogno, Marco Maria Fontanella, Liverana Lauretti, Diego Mazzatenta, Francesco Doglietto, Alberto Iannalfi

**Affiliations:** 1 Department of Neurosurgery, Fondazione Policlinico Universitario Agostino Gemelli IRCCS, Rome, Italy; 2 Faculty of Medicine and Surgery, Università Cattolica del Sacro Cuore, Rome, Italy; 3 Programma Neurochirurgia Ipofisi – Pituitary Unit, IRCCS Istituto delle Scienze Neurologiche di Bologna, Bologna, Italy; 4 Department of Biomedical and Neuromotor Sciences (DIBINEM), University of Bologna, Bologna, Italy; 5 Radiation Oncology Unit, Clinical Department, C.N.A.O (National Center for Oncological Hadrontherapy), Pavia, Italy; 6 Department of Internal Medicine and Therapeutics, University of Pavia, Pavia, Italy; 7 Neurosurgery, Department of Surgical Specialties, Radiological Sciences, and Public Health, University of Brescia, Brescia, Italy; 8 ENT Department, Bellaria Hospital AUSL di Bologna, Bologna, Italy; 9 Department of Neurosurgery, Mater Olbia Hospital, Olbia, Italy

**Keywords:** Craniopharyngioma, Endoscopic endonasal, Hypothalamic syndrome, Proton radiotherapy, Fractionated radiotherapy

## Abstract

**Background:**

Craniopharyngiomas are rare, benign tumors whose relationship with crucial neurovascular structures poses a significant surgical challenge. The endoscopic endonasal approach has improved outcomes, but in cases of optic pathways or hypothalamic involvement, complete resection is impossible without significant functional deficits. Subtotal resection followed by radiation therapy has been achieving comparable rates of tumor control to gross total resection alone. Proton therapy (PRT) achieves comparable oncological outcomes to photon therapy, but with better functional outcomes thanks to its capability of sparing surrounding healthy tissues. Herein we summarize the most recent evidence on the use of PRT for craniopharyngiomas and provides paradigmatic, challenging case scenarios managed with a combination of surgery and PRT.

**Methods:**

Eight patients who were treated with PRT for craniopharyngiomas between January 2016 and September 2023 are presented. Patients underwent thorough endocrinological, clinical, cognitive, and radiological evaluation before and after treatment.

**Results:**

All patients underwent at least one surgical procedure. One patient was treated with primary PRT. Five patients presented with visual impairment before treatment. All 8 cases had some degree of endocrinological dysfunction before PRT. Four patients presented signs of hypothalamic dysfunction. Seven patients received a conventional cycle of treatment (54 GyRBE total, 1.8 GyRBE/fraction, 30 fractions), but one patient received 52.5 GyRBE in 29 fractions with caution due to pre-treatment bitemporal hemianopia and old age. One patient’s visual acuity worsened within grade 2 (according to CTCAE v5.0), while all the others improved or remained stable after treatment. Endocrinological status improved in one case and remained stable in the others. Hypothalamic dysfunction worsened in two patients and improved in two. In one case, a complete response was achieved, in one the tumor remained stable, and the remaining six achieved a partial response with reduction ≥ 50%. A case with brain multifocal dissemination was successfully treated with surgery and systemic therapy, and concomitant asymptomatic early radio-induced contrast enhancement was observed. Median follow-up spanned 58.5 months (range: 24–72).

**Conclusions:**

PRT appears as a safe and feasible option for the management of craniopharyngiomas, especially for children and in cases of adherence to the optic pathways or hypothalamus, and generally in various challenging case scenarios. From a modern neuro-oncological perspective, multimodal function-sparing management should be favored over aggressive surgery.

**Supplementary Information:**

The online version contains supplementary material available at 10.1007/s10143-026-04258-1.

## Introduction

Craniopharyngiomas (CPs) are rare, benign tumors arising from remnants of Rathke’s pouch [1]. Their intimate relationship with the optic pathways, hypothalamic-pituitary axis, and major vessels of the brain poses a significant surgical challenge [2]. The increasing implementation of the extended endonasal endoscopic approach (EEA) has significantly increased rates of gross-total resection and decreased postoperative mortality [3]. This has led to fewer patients being referred to adjuvant therapy, with gross total resection (GTR) being the main driver of progression-free survival (PFS) [4]. It is argued that the increased surgical aggressiveness has led to increased rates of postoperative functional morbidities [5]. Recent evidence suggests that subtotal resection followed by radiation therapy (RT) achieves comparable oncological results to GTR alone, but with inferior rates of unfavorable functional outcomes [6, 7]. Proton radiotherapy (PRT) represents a safe and feasible choice for CPs thanks to its ability to preserve healthy tissue surrounding the target [8, 9] due to its physical selectivity and to the active scanning delivery methods. In particular, PRT permits sparing of significant cognitive function by minimizing the radiation dose to brain volumes and by more effectively performing the hippocampi-sparing approach [8–11], which makes it the ideal advanced radiation technology for CPs.

Herein, we provide an update of current evidence on PRT for CPs, as well as a description of paradigmatic cases from a referral center for PRT in brain tumors.

## Methods

This research was carried out according to PROCESS criteria [12]. The study was approved by the Gemelli Institutional Ethical Committee. Patient consent was collected at the time of treatment. Data on patients consecutively treated between January 2016 and September 2023 at the Radiation Oncology Unit of the National Center for Oncological Hadrontherapy (CNAO) in Pavia were retrospectively reviewed.

Patients’ clinical and radiological features were collected from institutional databases. All patients underwent comprehensive pre- and post-PRT endocrinological and visual assessment. BMI was assessed before PRT and at each subsequent checkup. Cognitive evaluation was based on the evaluation of physicians or on reports from patients’ relatives. Parameters evaluated to assess cognitive status included both long and short memory deficits, behavioral changes, hypothalamic hyperphagia, persistent confusion, reduced attention span, impaired executive functions. Case 8 was referred to a neuropsychologist. MRIs were performed before and after surgery, 1 month thereafter, and then every 6 or 12 months based on the presence of eventual residue/recurrence. Each patient underwent an MRI before PRT and 1 month after treatment, every 4 months for the first 2 years, and every 6 months after the third year. PRT response was qualitatively reported according to RECIST criteria [13], and radiological response was volumetrically measured and reported as a percentage of the initial volume at treatment. All outcomes are reported according to the last available evaluation. Tables 1 and 2 provide extended data for patients included in the study.

**Table 1:** Extended pretreatment and treatment data (ACP = adamantinomatous; BH = bitemporal hemianopia; HC = hypocortisolism; HGn = hypogonadism; HT = hypothiroidism; HyperPRL = hyperprolactinemia; IV = intraventricular; PCP = papillary; PHP = panhypopituitarism; RICE = radio-induced contrast enhancement; VI = reduced visual acuity)

Case no.	Age at PRT (years), sex	Treatment before PRT (time to PRT, months)	PrePRT visual symptoms	PrePRT CN deficit	PrePRT endocrine deficit	PrePRT hypothalamic dysfunction	Histology	Tumor location	Tumor consistency	Stalk resection at surgery	Hypothalamic infiltration at surgery	PRT total dose, fraction dose (fractions no.)	PRT complications
1	40, F	Ventriculoscopic cyst fenestration and biopsy (6)	None	None	HyperPRL	Obesity	PCP	Suprasellar/IV	Mixed	No	Yes	54, 1.8 (30)	None
2	58, F	EEA (40)	Inferior temporal hemianopia	Trigeminal neuropathy	HC, DI	Overweight	ACP	Suprasellar/IV + prepontine	Cystic	Yes	No	54, 1.8 (30)	None
3	50, M	EEA, TCA (1)	VI, left inferior temporal quadrantanopia	Left optic neuropathy	HC, HT, HGn, DI	None	ACP	Suprasellar	Cystic	Yes	Yes	54, 1.8 (30)	None
4	59, M	EEA (26)	None	None	PHP, DI	Obesity, short memory loss, disorientation	ACP	Suprasellar/IV	Cystic	Yes	No	54, 1.8 (30)	None
5	50, M	EEA, TCA (30)	Left homonimous hemianopia	Left optic neuropathy	PHP, DI	Cognitive decline	ACP	Suprasellar	Mixed	Yes	No	54, 1.8 (30)	None
6	56, F	EEA (8)	BH	None	PHP, DI	None	PCP	Suprasellar/IV	Solid	Yes	Yes	54, 1.8 (30)	RICE Grade 1
7	70, M	EEA (5)	BH	None	PHP, DI	None	PCP	Suprasellar	Mixed	Yes	Yes	52.5, 1.8 (29)	None
8	12, F	TCA, EEA (25)	None	None	PHP, DI	None	ACP	Suprasellar/IV + prepontine	Solid	Yes	Yes	54, 1.8 (30)	None

**Table 2:** Extended posttreatment and outcome data (OCD: obsessive compulsive disorder; PR: partial response)

Case no.	PostPRT visual symptosm	PostPRT CN deficit	PostPRT endocrinological dysfunction	PostPRT hypothalamic disfunction	Tumor response (%)	Recurrence/progression (time to recurrence, years)	PostPRT treatment	Follow-up (months)
1	None	None	Improved	Improved	PR (50%)	No	No	24
2	Improved	Improved	Stable	Improved	PR (> 50%)	No	No	25
3	Worsened	Worsened	Stable	None	PR (> 90%)	No	No	40
4	None	None	Stable	Worsened obesity	PR (> 50%)	No	No	42
5	Stable	Stable	Stable	Stable	Stability	No	No	75
6	NA	NA	NA	NA	Nodule necrosis	Out of RT field Intracranial dissemination (< 1)	Surgery + BRAF inhibitors	96
7	Improved	None	Stable	None	Complete (100%)	No	No	84
8	None	None	Stable	OCD, mixed depression-anxiety syndrome	PR (> 90%)	No	No	72

## Results

### Case 1 – mixed surgical-PRT management of heterogeneous CP

**Fig. 1:**
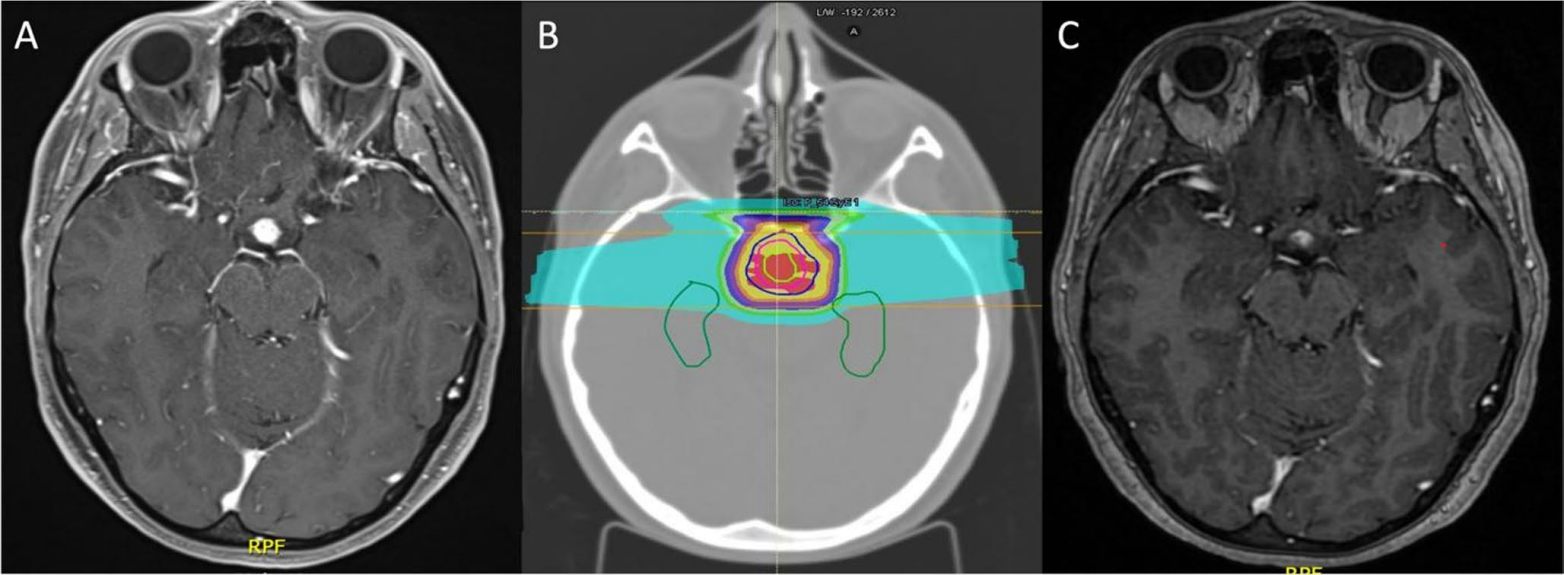
**A**, Axial view of nodular tumor right behind the optic chiasm between the optic tracts. **B**, Proton radiotherapy plan with a hippocampi-sparing approach (less than 40% of hippocampi receiving 7.3 GyRBE). **C**, Post-treatment scan showing signs of remission as soon as 3 months post-treatment

Patient 1 is a 40 year-old female who underwent primary PRT on a mixed solid-cystic-microcystic CP. Patients presented with hyperPRL and moderate obesity (BMI = 32 kg/m^2^). Pretreatment MRI showed a heterogeneous suprasellar/IV mass. The patient was subjected to a ventriculoscopic fenestration of the largest cystic component to minimize contact surface between the brain and tumor before PRT, optimizing the hippocampal-sparing approach. During the procedure, tissue specimens were collected and revealed a PCP. At 6 months after surgery, the patient underwent PRT on the residual nodule and microcystic components of the tumor. No complications were recorded during or after treatment, and the patient experienced a reduction in PRL levels and BMI (now within normal range). At the last radiological assessment, 24 months after the end of treatment, the tumor shrank and PR = 50% was obtained) (Fig. 1).

### Case 2 – PRT on a bifocal cystic recurrence with increased surgical risk

**Fig. 2:**
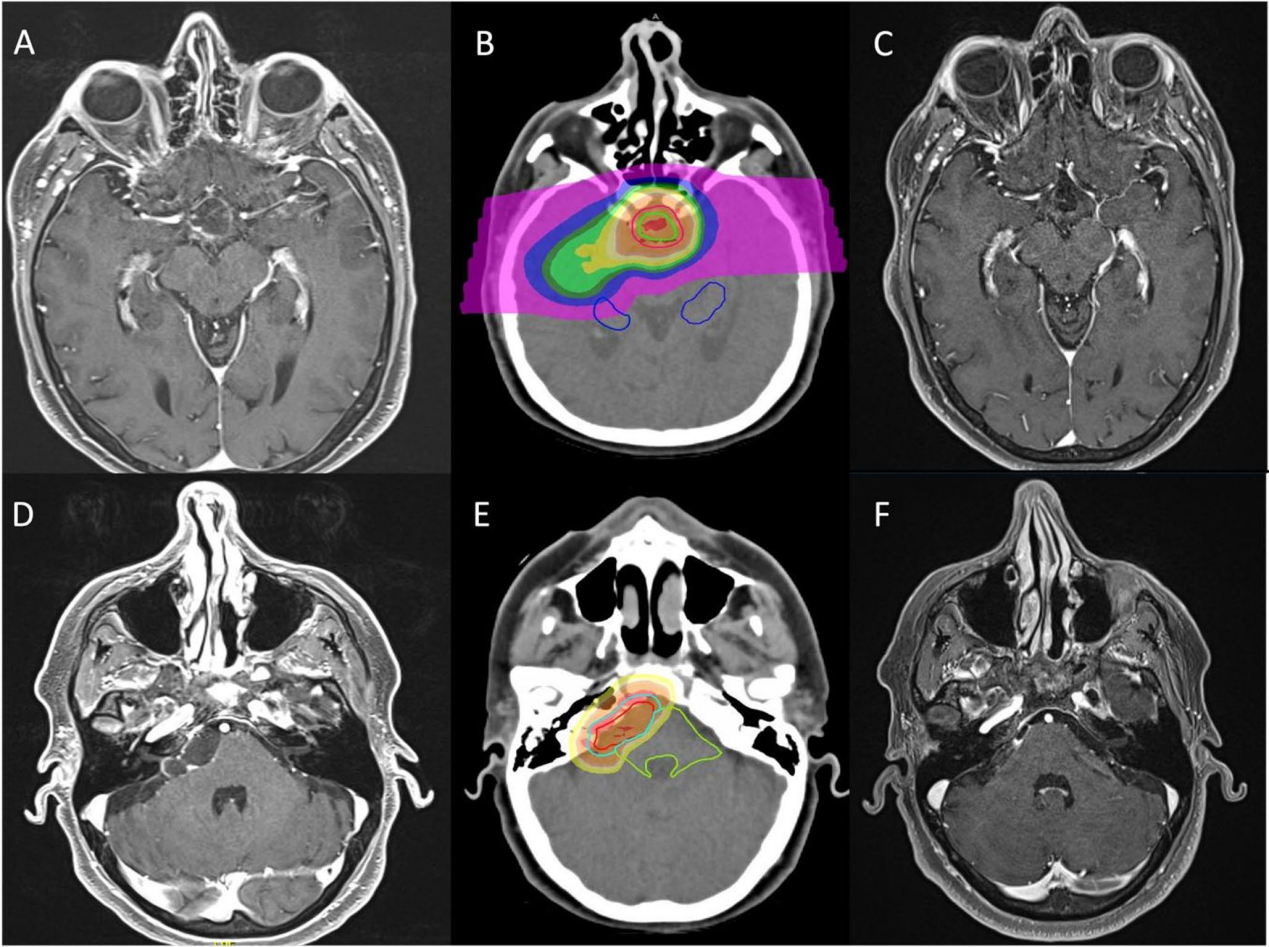
**A**, axial scan showing the recurrent cystic suprasellar component. **B**, PRT plan with hippocampi dose constraint respected (less than 40% of hippocampi volume receiving 7.3 GyRBE). **C**, Two-year follow-up scan with partial response. **D**, Axial scan view of the right ponto-cerebellar angle (APC) location of cystic recurrence. **E**, PRT plan focused on the APC recurrence abutting to the brainstem. **F**, Two-year follow-up MRI showing partial response

A 58 year-old female was referred to PRT after a first radical EEA on an adamantinomatous CP (ACP). The patient experienced a bifocal recurrence, where one cyst occurred in the suprasellar space, adherent to the optic chiasm, and the other on the prepontine cistern. Given the increased surgical risk due to the presence of chiasmatic adherences from the previous EEA, the patient was referred to PRT. Before treatment, the patient was slightly overweight (BMI = 26.6 kg/m^2^), vision in her inferior temporal quadrants was impaired, and she suffered from hypocortisolism, diabetes insipidus (DI), and trigeminal neuralgia. The patient underwent a course of PRT, targeting both cysts, uneventfully. Twenty-five months after treatment, the patient’s visual field, weight, and neuralgia were improved, while endocrinological status remained stable. Both cysts regressed and obtained a partial response > 50% (Fig. 2).

### Case 3 – PRT on para-suprasellar pluri-cystic CP after progression

**Fig. 3:**
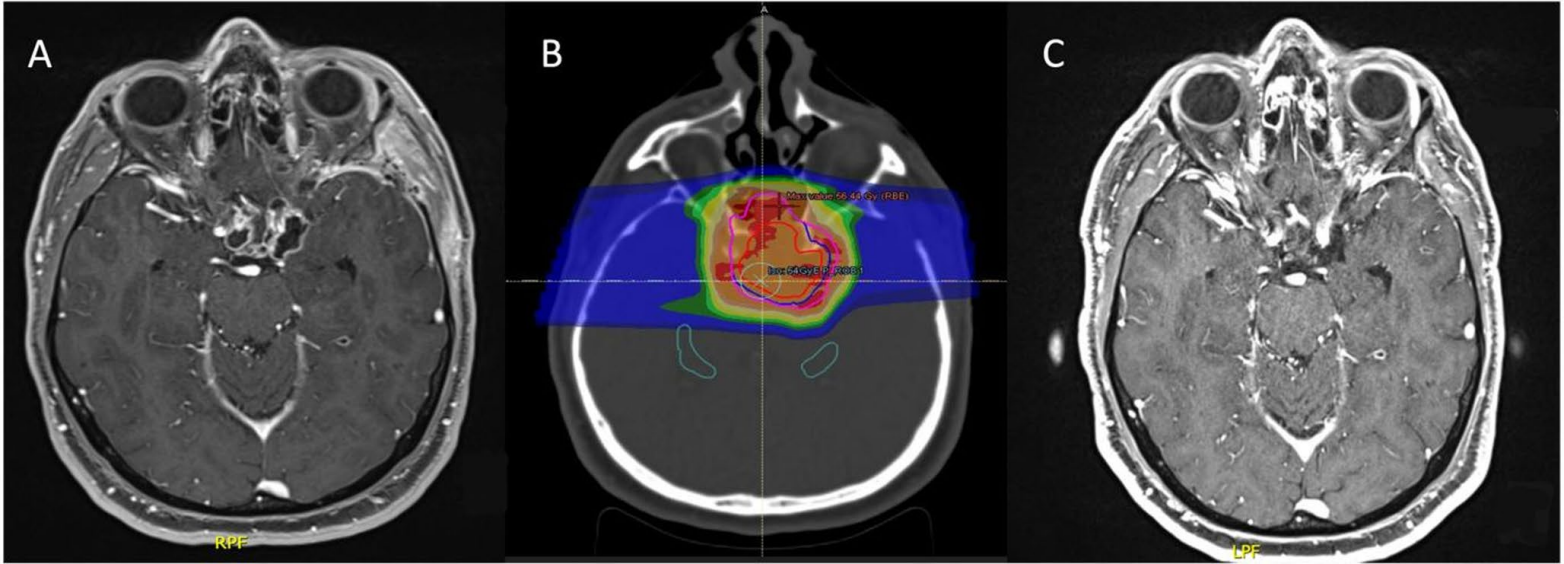
**A**, axial scan with residual CP in the sellar and right parasellar space. **B**, PRT isodose plan on multi-cystic target volumes and hippocampi-sparing approach. **C**, One-year follow-up scan revealing an almost complete response

The cavernous sinus is a forbidding surgical target. This patient, a 58-year-old male diagnosed with an ACP, underwent PRT after two surgeries. After a first subtotal resection via EEA for chiasmatic decompression, the parasellar-intracavernous residue was rapidly progressing as multicystic disease. The patient underwent a left pterional craniotomy to decompress the parasellar component, after which he was referred to radiation treatment. Before treatment, the patient presented with reduced visual acuity, inferior left quadrantanopia, and left optic neuropathy. He also exhibited hypocortisolism, hypothyroidism, hypogonadism, and DI. After a course of PRT, with no acute complications recorded, the pluri-cystic residue showed a near complete response (> 90%) that was stable at 40 months of follow-up. The patient’s visual function and optic neuropathy worsened, likely due to the close involvement of the visual apparatus by the tumor, but visual impairment remained within grade 2 (CTCAE v5.0). On the other hand, no damage to the cavernous carotid artery and/or cranial nerves was recorded (Fig. 3).

### Case 4 – treatment of a long-term recurrence adherent to the optic chiasm

Patient 4 is a 59-year-old male who received PRT at 26 months after a radical endoscopic surgery. Recurrence occurred strictly adhering to the optic chiasm, making surgery contraindicated. PrePRT findings included panhypopituitarism, DI, obesity (BMI = 37 kg/m^2^), and cognitive impairment with short-term memory loss and disorientation. The PRT adopted a conventional dose schedule (54 GyRBE in 30 fractions). Symptoms remained stable after treatment, except for body weight, which severely increased due to uncontrollable hyperphagia. The tumor exhibited a partial response (> 50%) after 42 months of follow-up.

### Case 5 – multi-recurrent CP controlled with PRT

This case is that of a 50-year-old male who had already undergone two surgeries (one EEA and one craniotomy) more than two years before radiation. A second recurrence occurred close to the optic chiasm, after which he was referred to PRT. He presented with left homonymous hemianopia, left optic neuropathy, panhypopituitarism, DI, obesity, and cognitive decline, all of which increased the surgical risk. After a conventional course of treatment (54 GyRBE in 30 fractions), none of the symptoms worsened, but the recurrence remained stable after 75 months of follow-up.

### Case 6 – intracranial dissemination after combined EEA plus PRT and radio-induced tumor intra-lesional necrosis

This is the case of a 56-year-old female who was operated via an EEA for a PCP. A subtotal resection was pursued due to the solid residue being adherent to the optic pathways and hypothalamus. The residue exhibited progression, and the patient was referred to PRT, when she presented with bitemporal hemianopia, panhypopituitarism, and DI. She underwent 30 fractions of PRT (1.8 GyRBE/fraction, 54 GyRBE total), after which she developed a grade 1 RICE (radiological finding with no clinical consequence). After less than a year from PRT, the irradiated primitive tumor nodule colliquated as a positive radio-induced response, but at the same time, the disease spread intracranially in a multifocal way with a major lesion at the level of the foramen magnum. Surgery was indicated for the foramen magnum dissemination (Ki-67 = 20%). After that, she sustained 8 years of BRAF inhibitor therapy (still ongoing) as the tumor exhibited the BRAFV600E mutation, with a complete response of all lesions except the infundibular one, which, after 96 months from PRT, is in partial remission, with 20% regression.

### Case 7 – complete response after PRT in an elderly patient with suprasellar CP

Patient 7 is a 70-year-old male who underwent a partial resection of a suprasellar PCP via EEA. Due to early progression of the nodule, which led to bitemporal hemianopia, PRT was indicated. The patient also presented with panhypopituitarism and DI. Because of pre-treatment visual impairment, older age, and close involvement of the optic chiasm, the PRT dose schedule chosen was 52.2 GyRBE in 29 fractions. Furthermore, the pre-treatment gross tumor volume occupied the third ventricle. No acute toxicity occurred. Nevertheless, after 84 months of follow-up, no sign of the original tumor is detectable, and the bitemporal hemianopia has resolved. After 5 years of follow-up, the patient experienced a right thalamic bleeding outside of the RT field with no clinical consequence.

### Case 8 – clinical scenario: aggressive brainstem CP in a child

**Fig. 4:**
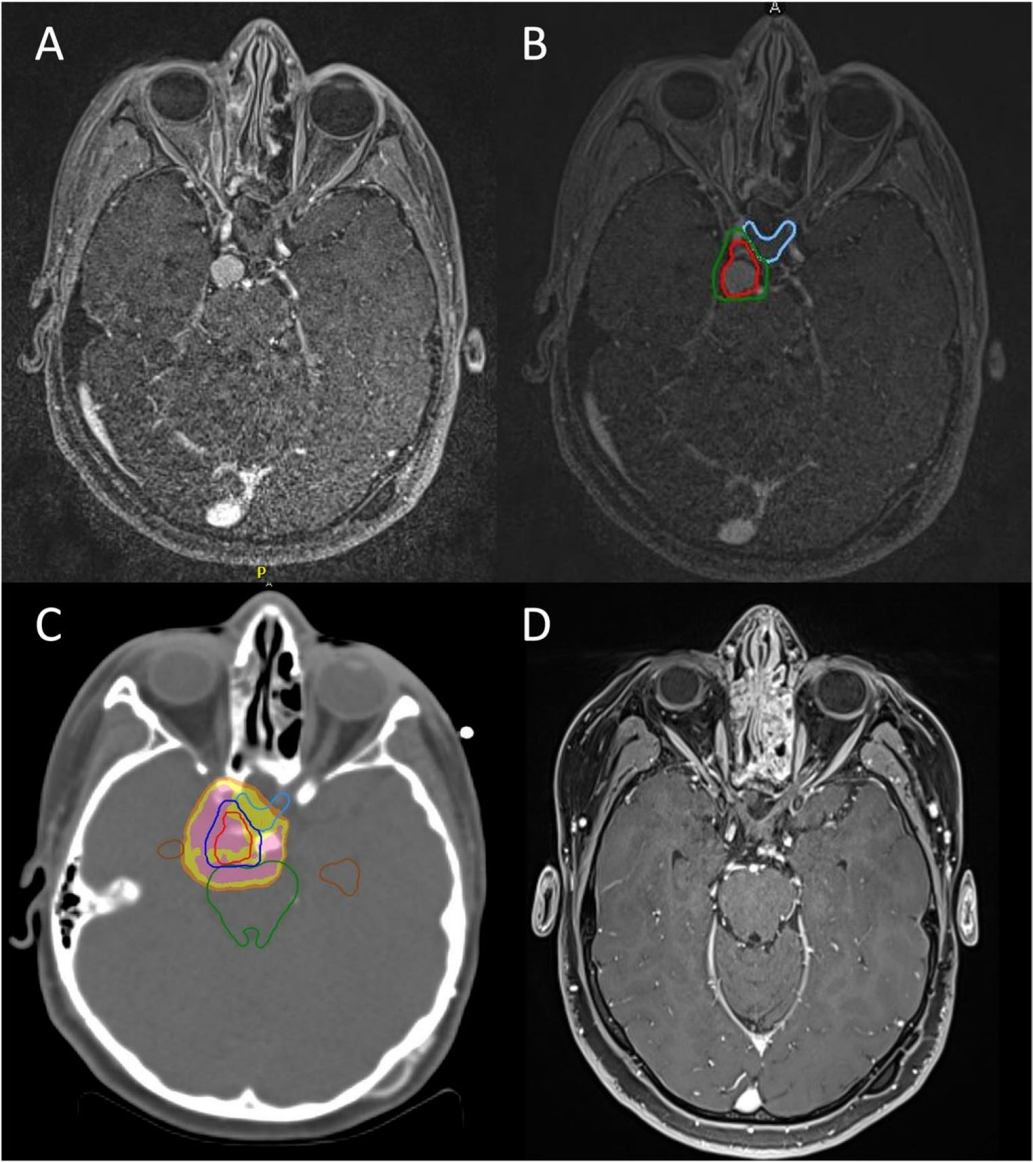
**A**, Axial view of nodular recurrence just below the right optic tract. **B**, PRT target delineation (gross tumor volume, red and clinical target volume, green) with optic chiasm (blue). **C**, PRT plan isodoses on target volume with brainstem (green) and chiasm delineation (blue). **D**, Five-year follow-up scan showing an almost complete response

Patient 8 is a 12-year-old girl diagnosed with an ACP presenting with a double cystic component (one in the third ventricle, and a larger inferior cyst extending into the left cerebellopontine angle). The patient presented with acute obstructive hydrocephalus and underwent urgent ventriculostomy, followed by a retrosigmoid craniotomy with fenestration of the cerebellopontine angle cyst and intracystic catheter placement. Three years later, progressive enlargement of the suprasellar component was documented. Given the neuroradiological growth and partial development of primary and secondary sexual characteristics, definitive surgical treatment was indicated (Video 1). The patient underwent an EEA resection, with residual adherent to the basilar artery and brainstem. Two years after the procedure, the residue started growing again, at which point PRT was indicated. Before treatment, she exhibited panhypopituitarism with DI. She underwent a conventionally fractioned schedule (54 GyRBE in 30 fractions). The residue showed early signs of regression, with the first two control MRIs revealing a gradual loss of contrast enhancement. At the last follow-up, 72 months from PRT, almost all the tumor had regressed, with less than 10% remaining. However, a neuropsychological assessment diagnosed her with anxious-depressive syndrome and obsessive–compulsive disorder (OCD) (Fig. 4).

## Discussion

### State of the art for PRT in CP and oncological outcomes

Given their benign histological grade (WHO-G1), the choice of RT modality for treatment of CPs must weigh in long-term local tumor control against functional preservation and minimization of toxicity. The current standard delivery technology in particle radiation is active scanning technology. This advanced technique allows different doses of radiation to be delivered to different regions within the tumor, minimizing damage to the surrounding healthy tissue [8, 11, 14–22]. Carbon ion RT should not represent a choice as particle radiation for treatment of CPs, given the higher RBE (relative-biological effectiveness: the ratio of absorbed doses to produce the same biological endpoint, using a reference radiation like photons, and the comparison type of radiation as particle [23]), which could lead to overtreatment in these cases. Carbon ion RT should be reserved for radioresistant tumors [23, 24]. Thus, PRT should be considered the unique particle radiation for CPs. PRT can be delivered in a conventionally fractioned schedule (1.8-2 GyRBE/fraction, 50–54 GyRBE total dose), which is the preferred option for large, complex tumors or in cases of tumors adjacent to optic pathways, cranial nerves, or vital neurological structures [21, 25, 26], features often encountered when facing CPs.

Despite their benign nature, recurrence of CPs is not rare [27], and GTR is the main predictor of PFS [4]. The increased employment of the EEA for CPs has led to increased rates of GTR, with inferior rates of progression/recurrences [5, 28, 29]. Nevertheless, GTR is often precluded by tumor-related factors - mainly hypothalamic invasion and/or strict adherence to the optic pathways. In these cases, patients are referred to radiation therapy to control residual/recurrent disease. The available data shows that rates of PFS are comparable between GTR and subtotal resection followed by radiation in both adults and children [6, 7]. Comparing photon and PRT, a recent meta-analysis [30] found that PRT achieves significantly superior tumor control compared to photon RT, where local control rates were respectively 98.8% vs. 92% at 3 years, and 92.7% vs. 81.8% at 5 years. The literature included in Table 3 reports local control rates of 91.7% − 94% after a median minimum follow-up of 2.7 years, while our experience shows a local control of 100% with a median follow-up of 58.5 months.

**Table 3:** Summary of studies cited in this paper (HC = hypocortisolism; HGn = hypogonadism; HT = hypothiroidism; NA: not available; NFFS: nodule-failure free survival)

First author, year	Patients (*n*)	Median age (years)	PRT total dose (fraction dose)	Local control (%)	Endocrinological outcomes	Hypothalamic outcomes	Visual outcomes	Toxicity	Follow-up (years)
Bischoff, 2025 [37]	65	9.1	54 (1.8)	NA	67.7% endocrinopathy	41.5% obesity	NA	NA	3.2
Merchant, 2024 [11]	94	9	54 (1.8)	93.6%	57.1% HC 79% HT 42.9% HGn 0% new DI	2% psychiatric disorders	7.4% visual impairment	2.1% necrosis 4.5% vasculopathy	7.5
Beddok, 2022 [22]	91	37	52.2–54 (1.8)	92%	2.4–46,7% range new pituitary axis defect	6% obesity 4.4% memory impairment	9.8% visual impairment	44% fatigue	3.3
Jimenez, 2021 [18]	77	8.6	54 (1.8)	92%	4% improved 7% new cases 47% worsened	No changes	10% improved 0% new cases 10% worsened	13% moya-moya	4.8
Ajithkumar, 2018 [14]	16	10.2	52.2–54 (1.8)	94%	29% endocrinopathy	7% insomnia	No changes	NA	29
Bishop, 2014 [34]	21	9.1	54 (1.8)	91.7% NFFS	76% enocrinopathy	19% obesity	5% visual impairment	10% vascular morbidity	33

Our series also reports one unique case of post-PRT intra-lesional necrosis of a solid BRAFV600E-mutated PCP, where the cells disseminated intracranially. Combination with BRAF inhibitors has again been confirmed to be another theoretically useful weapon against this subset of CPs [31], and the patient is in remission, both for the disseminated disease and for the infundibular mass treated with PRT.

The cystic component of most CPs is a predictor of worse outcomes [32]. Cysts tend to enlarge after radiation, often leading to a change in the treatment plan because they grow beyond the original volume [33]. Bishop et al. [34] found 33% of cases (*n* = 17) of immediate cyst growth (14 being transient) after PRT, and 27% of late growth. During PRT in cystic tumors, MRI surveillance is requested to adapt the target volume to inflammatory, radio-induced cyst enlargement [42]. After RT, it is difficult to codify progression criteria for cyst enlargement: in clinical experience, and this event is more often expected because of RT in the first-year follow-up. After the first year, in case of cyst enlargement, a tight follow-up is recommended, but only symptomatic, progressive enlargement suggests an actual progression.

During target volume delineation, a margin of 3–5 mm is usually added to gross tumor volume to obtain the clinical target volume, including potential areas of subclinical microscopic disease and changes of cystic components. This target volume delineation approach, especially in cases of cystic components, requires a conventionally fractionated dose schedule [11, 22, 35]. Furthermore, fractionated radiotherapy is suited for optimal dose distribution over complex-shaped target volumes.

### Functional outcomes and complications

When treating a benign tumor, functional outcomes matter as much as oncological ones. CPs pose a severe challenge to this objective given their intimate relationship with vascular, nervous, and endocrinological structures [36]. Pituitary deficits are widespread in patients with CP due to a multiplicity of factors, including both the disease and treatments. High rates of post-treatment pituitary dysfunction are not surprising, given that an already damaged gland is also included in the target volume for CP planning. Indeed, Bischoff et al. found that pre-treatment DI predicted the development of post-treatment anterior hypopituitarism [37]. In their series, all 65 patients presented at least one endocrinological axis deficiency at follow-up (median 3.2 years). Jimenez et al. reported a worsened endocrinological status in 47% of 77 patients, with 7% of them being newly diagnosed. However, DI rates fell to 12% post-PRT from 36% pre-PRT [18]. Beddok et al., among pre-PRT asymptomatic patients, observed a 2.4–46.7% range of post-PRT new pituitary axis defect [22]. In our experience, 5 of 8 patients presented panhypopituitarism, and 2 had more than one hormone deficiency before PRT, all of whom remained stable after treatment. Compared to other forms of RT, PRT has been described to determine similar rates of endocrinopathy, despite heterogeneity being reported across studies [11, 18, 34].

Regarding metabolic outcomes, Bischoff et al. found that BMI increased more between surgery and PRT, compared to after PRT, with hypothalamic involvement significantly correlated to BMI increase [37], suggesting that an already damaged hypothalamus is more likely to suffer from radiation.

The optic apparatus is also often included in the treatment, given its close vicinity to CP localizations. Moreover, surgeons are reluctant to aggressively dissect tumors from the optic nerves or chiasm. Thus, treatment volumes almost always must balance the radiation delivered to the apparatus in planning, and radiosurgical techniques are usually unfit given the higher dose of radiation conveyed to surrounding structures [38]. Indeed, most of our patients were irradiated on residual/recurrent lesions strictly adherent to the chiasm. PRT delivered in a conventional schedule has been shown to be respectful of the healthy optic tissues, which usually benefit from the regression of the tumor more than the detrimental effects of radiation as highlighted in large series. A conventionally fractionated dose schedule represents the indicated choice when tumors abut or are closely involved with optic pathways [21]. Interestingly, in Bishop’s series [34], 6 of the 8 patients whose vision worsened presented severe visual impairment pre-treatment, suggesting that pre-existing damage to the optic pathways predicts a worse visual outcome. The same evidence can be found in our series, where the only patient whose vision worsened had severe visual impairment pre-treatment (case 3). Accordingly, the available data shows a 5%-10% rate of visual worsening post-RT, as shown in Table 3.

The same considerations stand for neuropsychological outcomes. This is especially important in younger patients, where PRT represents the radiation of choice thanks to its better preservation of cognitive function [39]. Thus, in cases of hypothalamic infiltration, it is recommended to opt for subtotal resection followed by adjuvant therapy [40]. Radiological studies have shown that deep white matter declined early during the first post-PT year, but later exhibited recovery, except when the corpus callosum was damaged, in which case a complete recovery was not possible [41]. Uh et al. also revealed that declined cognitive function pre-treatment depended on damaged white matter tracts, and that surgery, obstructive hydrocephalus, and preoperative hypothalamic involvement were the main determinants of the damage. They also found a 5-year trend for white matter repair and cognitive efficiency, especially in children [42]. This is crucial to preserving intellectual abilities in young patients. PRT has been proven to more effectively spare neurocognitive functions thanks to the lower dose delivered to the hippocampi and brain [43], especially with the latest hippocampi-sparing approaches [44, 45]. This is particularly important in children and young adults, where hippocampal damage can lead to severe cognitive impairment and intellectual development retardation. PRT has been shown to better spare the hippocampi and brain areas linked to cognitive function, compared to photon RT. Merchant et al., in a phase 2 trial, found better cognitive outcomes in pediatric and adolescent patients affected by CPs who received PRT compared to historical photon RT phase II series [8–11, 46, 47]: PRT determines a significant difference for preservation of IQ and adaptive behavior [11]. Furthermore, even if their series comprised only children, Jimenez reported no cases of cognitive decline, but adaptive skills were decreased at follow-up although not in a clinically significant way [18]. Indeed, patient 8 developed OCD and anxious-depressive syndrome. Similarly, in adult patients, Beddok et al. have found a 96.7% rate of cognitive preservation and 95.6% of memory preservation in a recent large series [22].

Vascular complications are a main concern in radiation treatment for CPs given their usual intimate relationship with major arterial vessels of the brain. In children, it has also been related to the development of moya-moya syndrome, with rates ranging from 6% to 10% [18, 34]. Pre-PRT vasculopathy and proton dose to intact vessels have been linked to vascular stenosis [48]. Indeed, Yoo et al. have found a 33% prevalence of post-photon radiotherapy intracranial bleeding, with rates proportional to patient age [49]. We observed one case of thalamic bleeding (case 7), in areas not receiving a radiation dose out of the PRT field, likely due to the patient’s older age and comorbidities. We observed one case of RICE to parenchyma (case 6), with no clinical consequence.

## Limitations

The retrospective nature of the data presented herein carries some unavoidable bias. The limited population, and its heterogeneity, prevents us from elaborating significant statistical validity. Cognitive evaluation relies on physicians evaluation in 7 out of 8 cases, possibly introducing some bias. Nevertheless, we believe that this series provides some useful insights for clinicians, and the alignment of results from our series with the literature presented strengthens the validity of our claims.

## Conclusions

PRT appears as a safe and feasible option for cases of residual or recurrent CPs thanks to its high rates of tumor control and low toxicity. Especially in young patients, hippocampi-sparing techniques allow for optimal preservation of cognitive abilities. In adults, PRT is useful in cases requiring a conventional fractionated dose schedule, who are unsuitable for radiosurgery, and with tumors in proximity to the optic pathways. Overall, every skull base surgeon should be aware of this treatment option to properly manage CPs from a modern neuro-oncological point of view: a multimodal, function-sparing treatment modality should be favored against aggressive total resection at the cost of visual, endocrinological, and neurocognitive detriment.

## Supplementary Information

Supplementary Material 1. Case presentation of patient 8, including preoperative, intraoperative, and PRT description. Displaying the video on a 3D-compatible screen allows for 3D visualization while wearing 3D goggles

## Data Availability

No datasets were generated or analysed during the current study.

## Abbreviations

ACP: Adamantinomatous craniopharyngioma
BH: Bitemporal hemianopia
BMI: Body mass index
CP: Craniopharyngioma
CTCAE v5.0: Common terminology criteria for adverse events version 5.0
DI: Diabetes insipidus
EEA: Endoscopic endonasal approach
GTR: Gross-total resection
Gy(RBE): Gray (relative biological effectiveness)
HC: Hypocortisolism
HGn: Hypogonadism
HT: Hypothyroidism
IV: Intraventricular
OCD: Obsessive compulsive disorder
PCP: Papillary craniopharyngioma
PFS: Progression-free survival
PHP: Panhypopituitarism
PRL: Prolactinemia
PR: Partial response
PRT: Proton radiotherapy
RICE: Radio-induced contrast enhancement
RT: Radiotherapy
TCA: Transcranial approach
VI: Reduced visual acuity

## References

[1] Prabhu VC, Brown HG (2005) The pathogenesis of craniopharyngiomas. Childs Nerv Syst 21(8–9):622–627. 10.1007/s00381-005-1190-9 15965669 10.1007/s00381-005-1190-9

[2] Müller HL (2013) Paediatrics: surgical strategy and quality of life in craniopharyngioma. Nat Rev Endocrinol 9(8):447–449. 10.1038/nrendo.2013.125 23797818 10.1038/nrendo.2013.125

[3] Jeswani S, Nuño M, Wu A et al (2016) Comparative analysis of outcomes following craniotomy and expanded endoscopic endonasal transsphenoidal resection of craniopharyngioma and related tumors: a single-institution study. J Neurosurg 124(3):627–638. 10.3171/2015.3.JNS142254 26361276 10.3171/2015.3.JNS142254

[4] Ordóñez-Rubiano EG, Forbes JA, Morgenstern PF et al (2019) Preserve or sacrifice the stalk? Endocrinological outcomes, extent of resection, and recurrence rates following endoscopic endonasal resection of craniopharyngiomas. J Neurosurg 131(4):1163–1171. 10.3171/2018.6.JNS18901 30497145 10.3171/2018.6.JNS18901

[5] Nie C, Ye Y, Wu J, Zhao H, Jiang X, Wang H (2022) Clinical Outcomes of Transcranial and Endoscopic Endonasal Surgery for Craniopharyngiomas: A Single-Institution Experience. Front Oncol 12:755342. 10.3389/fonc.2022.755342 35223463 10.3389/fonc.2022.755342 PMC8866852

[6] Clark AJ, Cage TA, Aranda D et al (2013) A systematic review of the results of surgery and radiotherapy on tumor control for pediatric craniopharyngioma. Childs Nerv Syst 29(2):231–238. 10.1007/s00381-012-1926-2 23089933 10.1007/s00381-012-1926-2

[7] Dandurand C, Sepehry AA, Asadi Lari MH, Akagami R, Gooderham P (2018) Adult Craniopharyngioma: Case Series, Systematic Review, and Meta-Analysis. Neurosurgery 83(4):631–641. 10.1093/neuros/nyx570 29267973 10.1093/neuros/nyx570

[8] Boehling NS, Grosshans DR, Bluett JB et al (2012) Dosimetric comparison of three-dimensional conformal proton radiotherapy, intensity-modulated proton therapy, and intensity-modulated radiotherapy for treatment of pediatric craniopharyngiomas. Int J Radiat Oncol Biol Phys 82(2):643–652. 10.1016/j.ijrobp.2010.11.027 21277111 10.1016/j.ijrobp.2010.11.027

[9] Beltran C, Roca M, Merchant TE (2012) On the benefits and risks of proton therapy in pediatric craniopharyngioma. Int J Radiat Oncol Biol Phys 82(2):e281–287. 10.1016/j.ijrobp.2011.01.005 21570209 10.1016/j.ijrobp.2011.01.005 PMC3554244

[10] Dutz A, Lühr A, Troost EGC et al (2021) Identification of patient benefit from proton beam therapy in brain tumour patients based on dosimetric and NTCP analyses. Radiother Oncol 160:69–77. 10.1016/j.radonc.2021.04.008 33872640 10.1016/j.radonc.2021.04.008

[11] Merchant TE, Hoehn ME, Khan RB et al (2023) Proton therapy and limited surgery for paediatric and adolescent patients with craniopharyngioma (RT2CR): a single-arm, phase 2 study. Lancet Oncol 24(5):523–534. 10.1016/S1470-2045(23)00146-8 37084748 10.1016/S1470-2045(23)00146-8 PMC10408380

[12] Riaz AA, Ginimol M, Rasha R et al (2025) Revised Preferred Reporting of Case Series in Surgery (PROCESS) Guideline: An Update for the Age of Artificial Intelligence. Premier J Sci 2. 10.70389/PJS.100080

[13] Schwartz LH, Litière S, de Vries E et al (2016) RECIST 1.1-Update and clarification: From the RECIST committee. Eur J Cancer 62:132–137. 10.1016/j.ejca.2016.03.081 27189322 10.1016/j.ejca.2016.03.081 PMC5737828

[14] Ajithkumar T, Mazhari AL, Stickan-Verfürth M et al (2018) Proton Therapy for Craniopharyngioma - An Early Report from a Single European Centre. Clin Oncol (R Coll Radiol) 30(5):307–316. 10.1016/j.clon.2018.01.012 29459099 10.1016/j.clon.2018.01.012

[15] Bachtiary B, Veraguth D, Roos N et al (2022) Hearing Loss in Cancer Patients with Skull Base Tumors Undergoing Pencil Beam Scanning Proton Therapy: A Retrospective Cohort Study. Cancers (Basel) 14(16):3853. 10.3390/cancers14163853 36010847 10.3390/cancers14163853 PMC9405884

[16] Combs SE, Kessel K, Habermehl D, Haberer T, Jäkel O, Debus J (2013) Proton and carbon ion radiotherapy for primary brain tumors and tumors of the skull base. Acta Oncol 52(7):1504–1509. 10.3109/0284186X.2013.818255 23962241 10.3109/0284186X.2013.818255

[17] El Shafie RA, Czech M, Kessel KA et al (2018) Clinical outcome after particle therapy for meningiomas of the skull base: toxicity and local control in patients treated with active rasterscanning. Radiat Oncol 13(1):54. 10.1186/s13014-018-1002-5 29587795 10.1186/s13014-018-1002-5 PMC5870393

[18] Jimenez RB, Ahmed S, Johnson A et al (2021) Proton Radiation Therapy for Pediatric Craniopharyngioma. Int J Radiat Oncol Biol Phys 110(5):1480–1487. 10.1016/j.ijrobp.2021.02.045 33662460 10.1016/j.ijrobp.2021.02.045

[19] Murray FR, Snider JW, Bolsi A et al (2017) Long-Term Clinical Outcomes of Pencil Beam Scanning Proton Therapy for Benign and Non-benign Intracranial Meningiomas. Int J Radiat Oncol Biol Phys 99(5):1190–1198. 10.1016/j.ijrobp.2017.08.005 28939227 10.1016/j.ijrobp.2017.08.005

[20] Rutenberg MS, Rotondo RL, Rao D et al (2020) Clinical outcomes following proton therapy for adult craniopharyngioma: a single-institution cohort study. J Neurooncol 147(2):387–395. 10.1007/s11060-020-03432-9 32086697 10.1007/s11060-020-03432-9

[21] Li PC, Liebsch NJ, Niemierko A et al (2019) Radiation tolerance of the optic pathway in patients treated with proton and photon radiotherapy. Radiother Oncol 131:112–119. 10.1016/j.radonc.2018.12.007 30773177 10.1016/j.radonc.2018.12.007

[22] Beddok A, Scher N, Alapetite C et al (2023) Proton therapy for adult craniopharyngioma: Experience of a single institution in 91 consecutive patients. Neuro Oncol 25(4):710–719. 10.1093/neuonc/noac210 36002321 10.1093/neuonc/noac210 PMC10076942

[23] Iannalfi A, Riva G, Ciccone L, Orlandi E (2023) The role of particle radiotherapy in the treatment of skull base tumors. Front Oncol 13:1161752. 10.3389/fonc.2023.1161752 37350949 10.3389/fonc.2023.1161752 PMC10283010

[24] Gamez ME, Patel SH, McGee LA et al (2021) A Systematic Review on Re-irradiation with Charged Particle Beam Therapy in the Management of Locally Recurrent Skull Base and Head and Neck Tumors. Int J Part Ther 8(1):131–154. 10.14338/IJPT-20-00064.1 34285942 10.14338/IJPT-20-00064.1 PMC8270105

[25] Harrabi SB, Adeberg S, Welzel T et al (2014) Long term results after fractionated stereotactic radiotherapy (FSRT) in patients with craniopharyngioma: maximal tumor control with minimal side effects. Radiat Oncol 9:203. 10.1186/1748-717X-9-203 25227427 10.1186/1748-717X-9-203 PMC4261584

[26] Iannalfi A, Fragkandrea I, Brock J, Saran F (2013) Radiotherapy in craniopharyngiomas. Clin Oncol (R Coll Radiol) 25(11):654–667. 10.1016/j.clon.2013.07.005 23910225 10.1016/j.clon.2013.07.005

[27] Hussain I, Eloy JA, Carmel PW, Liu JK (2013) Molecular oncogenesis of craniopharyngioma: current and future strategies for the development of targeted therapies. J Neurosurg 119(1):106–112. 10.3171/2013.3.JNS122214 23560577 10.3171/2013.3.JNS122214

[28] Mortini P, Losa M, Pozzobon G et al (2011) Neurosurgical treatment of craniopharyngioma in adults and children: early and long-term results in a large case series. J Neurosurg 114(5):1350–1359. 10.3171/2010.11.JNS10670 21214336 10.3171/2010.11.JNS10670

[29] Calandrelli R, Pilato F, D’Apolito G et al (2025) Tumor features in adult papillary and adamantinomatous craniopharyngioma: neuroradiological evaluation of pituitary-hypothalamic-axis dysfunction and outcome prediction. Neuroradiology 67(5):1313–1327. 10.1007/s00234-025-03615-z 40293470 10.1007/s00234-025-03615-z

[30] Pan LS, Zhang J, Xiong YT, Zhan JB, Wang QY, Hong T (2025) Efficacy and safety of proton therapy and photon therapy for patients with craniopharyngioma: a systematic review and meta–analysis. Pituitary 28(5):103. 10.1007/s11102-025-01578-1 41006930 10.1007/s11102-025-01578-1 PMC12474669

[31] Fujio S, Ilmansyah R, Makino R et al (2025) Prospects of BRAF/MEK Inhibitor Therapy in Papillary Craniopharyngiomas with the BRAF V600E Mutation: A Scoping Review. Neurol Med Chir (Tokyo) 65(5):217–229. 10.2176/jns-nmc.2024-0246 40128998 10.2176/jns-nmc.2024-0246 PMC12137056

[32] Greenfield BJ, Okcu MF, Baxter PA et al (2015) Long-term disease control and toxicity outcomes following surgery and intensity modulated radiation therapy (IMRT) in pediatric craniopharyngioma. Radiother Oncol 114(2):224–229. 10.1016/j.radonc.2014.11.035 25542650 10.1016/j.radonc.2014.11.035

[33] Winkfield KM, Linsenmeier C, Yock TI et al (2009) Surveillance of craniopharyngioma cyst growth in children treated with proton radiotherapy. Int J Radiat Oncol Biol Phys 73(3):716–721. 10.1016/j.ijrobp.2008.05.010 18676089 10.1016/j.ijrobp.2008.05.010

[34] Bishop AJ, Greenfield B, Mahajan A et al (2014) Proton beam therapy versus conformal photon radiation therapy for childhood craniopharyngioma: multi-institutional analysis of outcomes, cyst dynamics, and toxicity. Int J Radiat Oncol Biol Phys 90(2):354–361. 10.1016/j.ijrobp.2014.05.051 25052561 10.1016/j.ijrobp.2014.05.051 PMC4194252

[35] Combs SE, Baumert BG, Bendszus M et al (2021) ESTRO ACROP guideline for target volume delineation of skull base tumors. Radiother Oncol 156:80–94. 10.1016/j.radonc.2020.11.014 33309848 10.1016/j.radonc.2020.11.014

[36] Gardner PA, Prevedello DM, Kassam AB, Snyderman CH, Carrau RL, Mintz AH (2008) The evolution of the endonasal approach for craniopharyngiomas. J Neurosurg 108(5):1043–1047. 10.3171/JNS/2008/108/5/1043 18447729 10.3171/JNS/2008/108/5/1043

[37] Bischoff M, Beckhaus J, Khalil DA et al (2025) Neuroendocrine Deficits and Weight Development Before and After Proton Therapy in Children With Craniopharyngioma. Clin Oncol (R Coll Radiol) 42:103837. 10.1016/j.clon.2025.103837 40239611 10.1016/j.clon.2025.103837

[38] Milano MT, Grimm J, Soltys SG et al (2021) Single- and Multi-Fraction Stereotactic Radiosurgery Dose Tolerances of the Optic Pathways. Int J Radiat Oncol Biol Phys 110(1):87–99. 10.1016/j.ijrobp.2018.01.053 29534899 10.1016/j.ijrobp.2018.01.053 PMC9479557

[39] Hess CB, Indelicato DJ, Paulino AC et al (2018) An Update From the Pediatric Proton Consortium Registry. Front Oncol 8:165. 10.3389/fonc.2018.00165 29881715 10.3389/fonc.2018.00165 PMC5976731

[40] Müller HL, Craniopharyngioma (2014) Endocr Rev 35(3):513–543. 10.1210/er.2013-1115 24467716 10.1210/er.2013-1115

[41] Uh J, Merchant TE, Li Y et al (2015) Effects of Surgery and Proton Therapy on Cerebral White Matter of Craniopharyngioma Patients. Int J Radiat Oncol Biol Phys 93(1):64–71. 10.1016/j.ijrobp.2015.05.017 26279025 10.1016/j.ijrobp.2015.05.017 PMC5144582

[42] Uh J, Merchant TE, Conklin HM et al (2021) Diffusion Tensor Imaging-Based Analysis of Baseline Neurocognitive Function and Posttreatment White Matter Changes in Pediatric Patients With Craniopharyngioma Treated With Surgery and Proton Therapy. Int J Radiat Oncol Biol Phys 109(2):515–526. 10.1016/j.ijrobp.2020.08.060 32898610 10.1016/j.ijrobp.2020.08.060

[43] Florijn MA, Sharfo AWM, Wiggenraad RGJ et al (2020) Lower doses to hippocampi and other brain structures for skull-base meningiomas with intensity modulated proton therapy compared to photon therapy. Radiother Oncol 142:147–153. 10.1016/j.radonc.2019.08.019 31522879 10.1016/j.radonc.2019.08.019

[44] Kahalley LS, Peterson R, Ris MD et al (2020) Superior Intellectual Outcomes After Proton Radiotherapy Compared With Photon Radiotherapy for Pediatric Medulloblastoma. J Clin Oncol 38(5):454–461. 10.1200/JCO.19.01706 31774710 10.1200/JCO.19.01706 PMC7007288

[45] Gross JP, Powell S, Zelko F et al (2019) Improved neuropsychological outcomes following proton therapy relative to X-ray therapy for pediatric brain tumor patients. Neuro Oncol 21(7):934–943. 10.1093/neuonc/noz070 30997512 10.1093/neuonc/noz070 PMC6620628

[46] Merchant TE, Hua C, ho, Shukla H, Ying X, Nill S, Oelfke U (2008) Proton versus photon radiotherapy for common pediatric brain tumors: Comparison of models of dose characteristics and their relationship to cognitive function. Pediatr Blood Cancer 51(1):110–117. 10.1002/pbc.21530 18306274 10.1002/pbc.21530

[47] Edmonston DY, Wu S, Li Y, Khan RB, Boop FA, Merchant TE (2022) Limited surgery and conformal photon radiation therapy for pediatric craniopharyngioma: long-term results from the RT1 protocol. Neuro Oncol 24(12):2200–2209. 10.1093/neuonc/noac124 35556133 10.1093/neuonc/noac124 PMC9713513

[48] Lucas JT, Faught AM, Hsu CY et al (2022) Pre- and Posttherapy Risk Factors for Vasculopathy in Pediatric Patients With Craniopharyngioma Treated With Surgery and Proton Radiation Therapy. Int J Radiat Oncol Biol Phys 113(1):152–160. 10.1016/j.ijrobp.2021.12.172 34990778 10.1016/j.ijrobp.2021.12.172 PMC9018579

[49] Yoo DH, Song SW, Yun TJ et al (2015) MR Imaging Evaluation of Intracerebral Hemorrhages and T2 Hyperintense White Matter Lesions Appearing after Radiation Therapy in Adult Patients with Primary Brain Tumors. PLoS ONE 10(8):e0136795. 10.1371/journal.pone.0136795 26322780 10.1371/journal.pone.0136795 PMC4556481

